# Epidemiology and Outcomes of Alcohol-Associated Hepatitis in Adolescents and Young Adults

**DOI:** 10.1001/jamanetworkopen.2024.52459

**Published:** 2024-12-27

**Authors:** Jennifer A. Flemming, Maya Djerboua, Orli Chapman, Oyedeji Ayonrinde, Norah A. Terrault

**Affiliations:** 1 Department of Medicine, Queen’s University Ontario, Ontario, Canada; 2 Department of Public Health Sciences, Queen’s University Ontario, Ontario, Canada; 3 ICES-Queen’s, Ontario, Canada; 4 Department of Psychiatry, Queen’s University Ontario, Ontario, Canada; 5 Department of Medicine, University of Southern California, Los Angeles

## Abstract

**Importance:**

Alcohol-associated hepatitis (AH) has high mortality, and rates are increasing among adolescents and young adults (AYAs).

**Objective:**

To define the sex-specific epidemiology of AH in AYAs and the association between female sex and liver-related outcomes after a first presentation of AH.

**Design, Setting, and Participants:**

A retrospective, population-based cohort study of routinely collected health care data held at ICES from Ontario, Canada, was conducted. Data on AYAs (age, 13-39 years) with first presentation of AH without a history of cirrhosis and/or decompensation from January 1 to December 31, 2022, were included in the analysis.

**Exposures:**

Study year and female sex.

**Main Outcome and Measures:**

Overall and sex-specific yearly rates of AH were compared using Poisson regression and rate ratios (RRs). Associations between female sex and incident cirrhosis and/or decompensation were evaluated using competing risks regression, and liver-related mortality by sex was evaluated with cumulative incidence functions.

**Results:**

A total of 3340 AYAs with AH were identified. Median age was 33 (IQR, 28-36) years, and the population included 1190 (36%) females and 2150 (64%) males. Rates of AH increased by 8% per year (RR, 1.08; 95% CI, 1.07-1.09), with yearly rates increasing faster among females (RR, 1.11; 95% CI, 1.09-1.12) than males (RR, 1.07; 95% CI, 1.06-1.07). A total of 2374 individuals (71%) were alive without cirrhosis 6 months after AH presentation. Of those, 527 (22%) developed incident cirrhosis and/or decompensation after a median follow-up of 4 (IQR, 2-9) years (37% females vs 29% males; *P* < .001). After adjustment, female sex was associated with a 47% higher subhazard of cirrhosis and/or decompensation compared with male sex (38%) (subhazard, 1.47; 95% CI, 1.23-1.76; *P* < .001). The cumulative incidence of liver-related mortality at 10 years was higher among females (11.0%; 95% CI, 8.3%-14.2%) than males (6.9%; 95% CI, 5.4%-8.6%) (*P* = .01).

**Conclusions and Relevance:**

Over the past 2 decades, the rates of AH among AYAs increased significantly, with the greatest increase observed among females. The findings of this study suggest sex-specific interventions to prevent the development of AH and the progression to cirrhosis after an episode of AH are needed.

## Key Points

**Question:**

Is sex associated with long-term liver-related outcomes among adolescents and young adults after first presentation of alcohol-associated hepatitis?

**Findings:**

In a cohort study of 3340 adolescents and young adults from Ontario, Canada, female sex was associated with a 50% higher risk of developing cirrhosis compared with male sex. The rate of liver-related mortality also was higher among females.

**Meaning:**

The findings of this study suggest that sex-specific interventions for alcohol-associated hepatitis among adolescents and young adults are urgently needed.

## Teaser

This cohort study examines mortality due to alcohol-associated hepatitis among adolescents and young adults in Canada, with findings stratified by sex.

## Introduction

Worldwide, alcohol is responsible for 13.5% of deaths among individuals aged 20 to 39 years.^1^ Alcohol-associated liver disease (ALD) is a spectrum from reversible hepatic steatosis to cirrhosis and liver failure with the most severe acute form of ALD being alcohol-associated hepatitis (AH).^2^ Alcohol-associated hepatitis has a 30-day mortality rate ranging from 30% to 50% for those with severe disease, and historic studies have shown AH is associated with a high risk of progression to cirrhosis in those who survive.^3^

Recent studies have consistently shown rates of AH are increasing.^4,5^ The reasons for these trends are not clear but hypothesized to be strongly correlated with governmental alcohol policies.^6^ Of most concern, adolescents and young adults (AYAs) (age, 13-39 years) have developed disproportionate increases in AH-related harms.^7^ The COVID-19 pandemic further exacerbated these trends with recent data both from the US^5^ and Canada^8^ showing increases in hospitalizations for AH, especially in young females.

Given the surging rates of AH among AYAs, there is an urgent need to identify risk groups and short- and long-term outcomes to facilitate targeted interventions and chronic disease management plans. Furthermore, sex-specific low-risk alcohol consumption guidelines recognize that, for the same amount of alcohol consumption, females are at higher risk of ALD compared with males^9^ due to biologic,^10^ cultural, and social factors. However, the influence of sex on the development of major liver-related outcomes, such as cirrhosis and decompensation, among AYAs with AH is understudied. Our objectives were to define the epidemiology and demographic characteristics of AYAs after an initial presentation of AH and evaluate the possible association between sex and long-term liver-related outcomes after an initial presentation of AH.

## Methods

### Study Design and Data Sources

We conducted a population-based retrospective cohort study in Ontario, Canada, from January 1 to December 31, 2022. This study used data from ICES, an independent, nonprofit research institute that houses a large electronic repository of datasets for all Ontario residents who are eligible for the province’s single-payer health care system (Ontario Health Insurance Plan).^11^ Due to the retrospective nature of this study, a waiver of informed consent was obtained from the Queen's University Health Sciences Research Ethics Board. This study used multiple administrative datasets (eTable 1 in Supplement 1), and these datasets were linked using unique encoded identifiers and analyzed at ICES. The study protocol received ethics approval from the Queen’s University Health Sciences Research Ethics Board and was conducted in accordance with the Strengthening the Reporting of Observational Studies in Epidemiology (STROBE) reporting guideline for observational studies.

### Study Populations

Adolescents and young adults aged 13 to 39 years with an incident diagnosis of AH were identified through inpatient hospital admissions and emergency department (ED) records. We used the *International Classification of Diseases, 9th Revision* (*ICD-9*) and *International Statistical Classification of Diseases and Related Health Problems, 10th Revision* (*ICD-10*) to identify AH as the main or most responsible diagnosis for the hospital admission or ED visit (*ICD-9* code 571.1; *ICD-10* code K70.1) associated with a positive predictive value for AH of 90%.^12^ If individuals had multiple AH presentations, the index event was used. Incident diagnoses were captured from January 1, 2002, to December 31, 2021, with a lookback window to the earliest available data and follow-up was until December 31, 2022. The type of AH presentation was defined as diagnosis and discharge from the ED vs hospital admission.

We excluded those who had our outcomes before the first AH presentation (cirrhosis and/or decompensation using validated definitions in ICES data; history of liver transplant (LT) identified through billing claims (eTable 2 in Supplement 1) and those with an invalid health card number and no Ontario Health Insurance Plan eligibility. We also identified a cohort at risk of outcomes if they survived their first presentation of AH by further excluding individuals who died or developed cirrhosis and/or decompensation during their initial AH presentation or within 6 months after ED or hospital discharge to exclude prevalent disease.

### Exposures, Outcomes, Demographics, and Covariates

Full details of definitions for exposures, outcomes, demographic characteristics, and covariates are in eTable 2 and the eMethods in Supplement 1. Sex was the primary exposure obtained from the Registered Persons Database. The outcomes of interest included all-cause mortality with death date obtained from the Registered Persons Database (up to December 31, 2022). Incident cirrhosis and/or decompensation (including ascites, variceal bleeding, encephalopathy, and hepatorenal syndrome) were defined using validated definitions.^13^ Liver transplant was identified using billing codes. Cause-specific mortality was defined to the end of 2018 using linkage to the Office of the Registrar General Death database (liver-related, external, mental health/behavioral, and other) (eTable 2 in Supplement 1).

### Statistical Analysis

Baseline characteristics were stratified by sex and type of presentation (hospital admission vs ED only). A denominator of AYAs in Ontario from 2002 to 2021 was built from the Registered Persons Database and used to calculate the overall and sex-stratified yearly incidence rates of AH with 95% CIs. Patients were considered at risk for incident AH until diagnosis of cirrhosis, receipt of LT, death, 40th birthday, loss of Ontario Health Insurance Plan coverage, and maximum study date (December 31, 2022). Poisson regression was used to evaluate the overall and sex-specific yearly changes of rates of AH over the study period using rate ratios (RRs).

Fine and Gray competing risk analysis was performed to assess the association of sex with each outcome as follows: all-cause mortality with LT as a competing event, and cirrhosis and/or decompensation with mortality and LT as competing events. For the competing risk analysis for overall mortality, follow-up started at the date of AH presentation and was adjusted a priori for age, type of presentation (inpatient vs ED only), cirrhosis during index AH presentation, Elixhauser comorbidity index, rural residence, immigrant or refugee status, and income quintile. For the competing risk analysis of the outcome of incident cirrhosis, follow-up started 6 months after the index and was adjusted for the same covariates except cirrhosis. Subdistribution hazard ratios (sHRs) with 95% CIs were computed to quantify the association between sex and outcomes.

Nonparametric competing risk regression was performed to estimate the overall and sex-stratified cumulative incidence function plots for liver-related mortality (with LT and non-liver deaths as competing events) in addition to all-cause mortality and cirrhosis. The Gray test was conducted to test for statistical differences between the sex-stratified cumulative incidences.^14^

All statistical findings were evaluated at a significance level of *P* < .05 with 2-sided, unpaired testing. All data were analyzed using SAS Enterprise Guide, version 7.1 (SAS Institute Inc).

## Results

### Study Population

**Figure 1.**
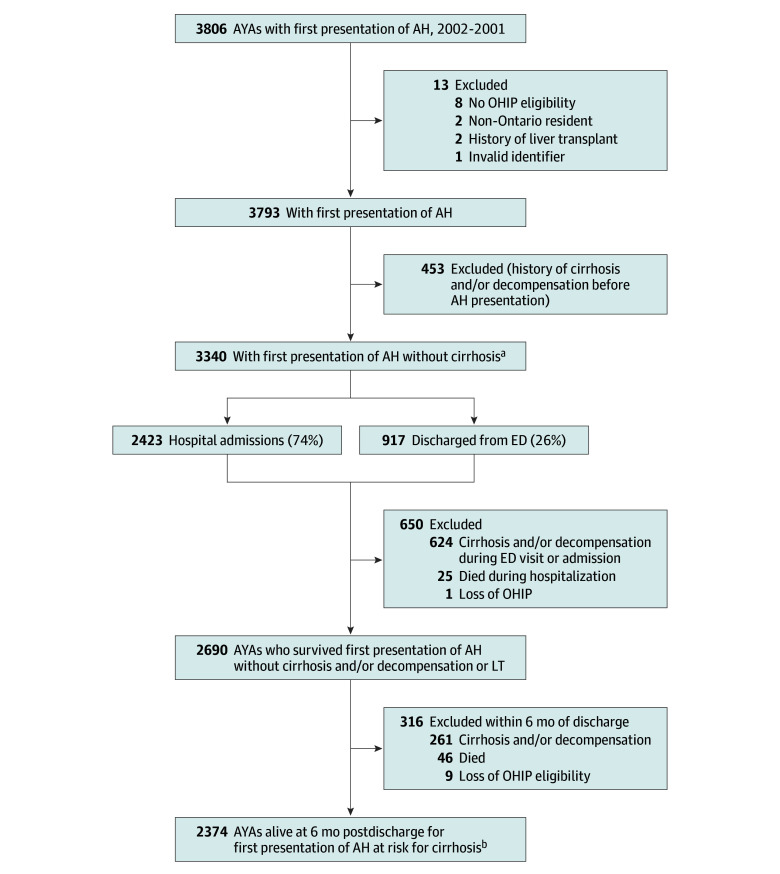
Development of a Cohort of Adolescents and Young Adults (AYAs) (Age, 13-39 Years) With Incident Alcohol-Associated Hepatitis (AH) From 2002-2021 ED indicates emergency department; LT, liver transplant; OHIP, Ontario Health Insurance Plan. ^a^Cohort used for overall and liver-related mortality outcomes. ^b^Cohort used for cirrhosis and/or decompensation outcomes.

Overall, 3340 AYAs were identified with first presentation of AH during the study and who did not have a history of cirrhosis or decompensation (Figure 1, Table 1). The median follow-up was 5 (IQR, 2-10) years and most required hospitalization (2423 [74%]) with a median length of stay of 4 (IQR, 3-8) days. The median age was 33 (IQR, 28-36) years, 1190 (36%) were female, 2150 (64%) were male, and 434 (13%) were immigrants or refugees with a median time from arrival in Ontario to AH of 14 (IQR, 9-21) years. Most resided in neighborhoods of the lowest income quintile (32%) and highest quintiles for material deprivation (27%) and residential instability (30%). Within 2 years before AH presentation, most (2365 [71%]) had ED or inpatient health care encounters related to the consequences of alcohol not due to AH, 1136 (34%) had encounters for other substance use, and over half (1912 [57%]) had encounters for mental health. Viral hepatitis was present in 8% (n = 282) and HIV in 0.6% (n = 19) of the AYAs. When stratified by sex, females vs males were more likely to reside in a rural location (22% vs 15%; *P* < .001) and have mental health encounters within 2 years of the index presentation (64% vs 53%; *P* < .001). More males were immigrants or refugees (16% vs 7%; *P* < .001) and resided in the most ethnically diverse neighborhoods (26% vs 17%; *P* < .001).

**Table 1.** Individuals in Ontario Aged 13 to 39 Years With First Presentation of Alcohol-Associated Hepatitis Without Known History of Cirrhosis, Decompensation, or LT: 2002-2021

Variable	No. (%)		
	Overall (N = 3340)	Females (n = 1190)	Males (n = 2150)
Age at presentation, median (IQR), y	33 (28-36)	32 (27-36)	33 (28-37)
Rural residence^a^	584 (18)	257 (22)	327 (15)
Missing	16 (0.5)	<6 (<0.5)	12 (0.5)
Recent immigrant/refugee^b^	434 (13)	86 (7)	348 (16)
History of ED visit/inpatient admission for alcohol^c^	2365 (71)	856 (72)	1509 (70)
History of mental illness^c^	1912 (57)	763 (64)	1149 (53)
History of substance use^c^	1136 (34)	416 (35)	720 (34)
Viral hepatitis	282 (8)	121 (10)	161 (7)
ECI			
0-2	3072 (92)	1084 (91)	1989 (93)
3-5	243 (7)	98 (8)	145 (7)
≥ 6	24 (1)	8 (1)	16 (1)
MELD-Na, median (IQR)^d^	15 (9-21)	14 (9-20)	15 (9-22)
<10	319 (30)	123 (29)	196 (30)
10-20	439 (40)	180 (43)	259 (39)
>20	321 (30)	114 (27)	207 (31)
Income quintile			
1 (Lowest)	1080 (32)	436 (37)	644 (30)
2	667 (20)	211 (18)	456 (21)
3	553 (17)	195 (16)	358 (17)
4	537 (16)	181 (15)	356 (17)
5 (Highest)	462 (14)	154 (13)	308 (14)
Missing	41 (1)	13 (1)	28 (1)
Material deprivation quintile			
1 (Lowest)	479 (14)	167 (14)	312 (15)
2	481 (14)	158 (13)	323 (15)
3	547 (16)	169 (14)	378 (18)
4	663 (20)	228 (19)	435 (20)
5 (Highest)	906 (27)	348 (29)	558 (26)
Missing	264 (8)	120 (10)	144 (7)
Ethnic diversity quintile			
1 (Lowest)	565 (17)	203 (17)	362 (17)
2	531 (16)	215 (18)	316 (15)
3	616 (19)	227 (19)	389 (18)
4	597 (18)	223 (19)	374 (17)
5 (Highest)	767 (23)	202 (17)	565 (26)
Missing	264 (8)	120 (10)	144 (7)
Residential instability quintile			
1 (Lowest)	454 (14)	120 (10)	334 (16)
2	448 (13)	137 (12)	311 (15)
3	539 (16)	202 (17)	337 (16)
4	618 (19)	246 (21)	372 (17)
5 (Highest)	1017 (30)	365 (31)	652 (30)
Missing	264 (8)	120 (10)	144 (7)
Follow-up time, median (IQR), y	5 (2-10)	5 (2-9)	5 (2-10)
Died anytime during follow-up	844 (25)	299 (25)	545 (25)
Liver transplant	23 (<1)	<6 (<1)	17-22 (<1)

Abbreviations: ECI, Elixhauser comorbidity index; ED, emergency department; LT, liver transplant; MELD-Na, model for end-stage liver disease.

^a^ Rural defined as location with less than 10 000 inhabitants.

^b^ Immigrant or refugee who entered Canada after 1985.

^c^ History within 2 years of alcohol-associated hepatitis presentation.

^d^ MELD-Na if available up to 7 days before or after presentation; missing values for 2261 individuals (68%).

At the time of AH, 624 individuals (19%) received diagnoses of cirrhosis or decompensation and a further 261 (7%) developed these conditions within 6 months, representing prevalent disease. Thus, 885 AYAs (26%) were considered to have cirrhosis or decompensation at index AH presentation. In those with available laboratory data within up to 7 days before or 7 days after AH presentation (1079 [32%]), the median model for end-stage liver disease (MELD-Na) score was 15 (IQR, 9-21), and 321 individuals (30%) had a MELD-Na score of 21 or higher, consistent with severe AH. The median MELD-Na score was higher among those hospitalized compared with those discharged from the ED (16 [IQR, 10-23] vs 9 [IQR, 7-13]). During follow-up, 25% AYAs died (n = 844) and less than 1% (n = 23) received LT.

### Epidemiology of AH in AYAs

**Figure 2.**
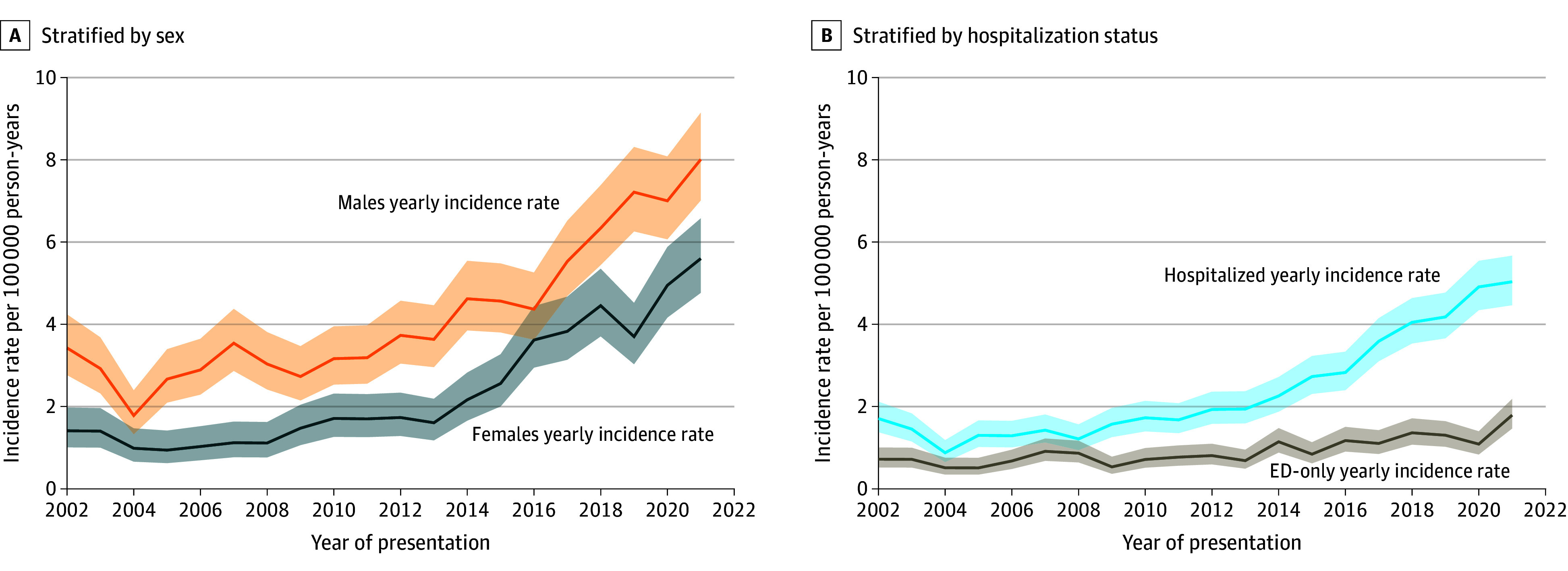
Incidence Rates of Alcohol-Associated Hepatitis Among Adolescents and Young Adults in Ontario Incidence rates stratified by sex (A) and hospitalization status (B). Shaded areas indicate 95% CIs. ED indicates emergency department; RR, rate ratio.

The annual incidence rate of first presentation of AH in AYAs increased from 2.43 per 100 000 person-years in 2002 to 6.82 per 100 000 person-years in 2021, corresponding to a yearly increase of 8% (RR, 1.08; 95% CI, 1.07-1.09; *P* < .001). We next stratified by sex over eras (Figure 2A), with rates increasing faster among females (RR, 1.11; 95% CI, 1.09-1.12) than males (RR, 1.07; 95% CI, 1.06-1.07). When stratified by hospitalization status (Figure 2B; eTable 3 in Supplement 1), rates of AH requiring hospitalization (RR, 1.09; 95% CI, 1.08-1.10) increased over the study period more than the rates of presentation to the ED only (RR, 1.05; 95% CI, 1.04-1.06).

### Overall and Liver-Associated Mortality Outcomes

The cumulative incidence of death at 1 year was 6.9% (95% CI, 6.2%-7.9%), 18.9% at 5 years (95% CI, 17.4%-20.3%), 29.4% at 10 years (95% CI, 27.5%-31.4%), and 47.7% at 20 years (95% CI, 43.5%-51.8%). When stratified by sex, there was no association with overall mortality (eFigure 1 in Supplement 1). Furthermore, in competing risks regression (Table 2), there was no association between female sex and overall mortality (sHR, 1.10; 95% CI, 0.95-1.27). Factors associated with mortality included older age (sHR per year, 1.03; 95% CI, 1.02-1.05), rural location (sHR, 0.81; 95% CI, 0.67-0.97), higher comorbidity (sHR, 1.99; 95% CI, 1.62-2.44), hospitalization at AH index (sHR, 1.40; 95% CI, 1.19-1.65), and cirrhosis during index AH presentation (sHR, 1.75; 95% CI, 1.48-2.07).

**Table 2.** Competing Risk Regression Analysis Evaluating the Association Between Sex and Overall Mortality Among 3340 Adolescents and Young Adults After First Presentation of Alcohol-Associated Hepatitis

Variable	Unadjusted		Adjusted	
	sHR (95% CI)	*P* value	sHR (95% CI)	*P* value
Female vs male sex	1.13 (0.98-1.31)	.08	1.10 (0.95-1.27)	.21
Age, y	1.04 (1.03-1.05)	<.001	1.03 (1.02-1.05)	<.001
Type of presentation				
ED only	1 [Reference]	NA	1 [Reference]	NA
Hospital admission	1.62 (1.38-1.91)	<.001	1.40 (1.19-1.65)	<.001
Cirrhosis at index	1.92 (1.64-2.26)	<.001	1.75 (1.48-2.07)	<.001
ECI				
0-2	1 [Reference]	NA	1 [Reference]	NA
≥3	2.06 (1.69-2.52)	<.001	1.99 (1.62-2.44)	<.001
Rural location	0.88 (0.74-1.04)	.14	0.81 (0.67-0.97)	.02
Recent immigrant/refugee	0.94 (1.64-2.26)	.52	0.91 (0.74-1.12)	.37
Income quintile				
1 (Lowest)	1.12 (0.90-1.40)	.45	1.09 (0.87-1.36)	.45
2	1.06 (0.84-1.34)	.67	1.05 (0.83-1.33)	.67
3	0.98 (0.76-1.26)	.87	0.98 (0.76-1.26)	.87
4	0.91 (0.70-1.18)	.30	0.87 (0.67-1.13)	.30
5 (Highest)	1 [Reference]	NA	1 [Reference]	NA

Abbreviations: ECI, Elixhauser comorbidity index; ED, emergency department; NA, not applicable; sHR, subdistribution hazard ratio.

Among those with available cause of death (to end 2018; n = 461 [55%]) (eFigure 2A in Supplement 1), the most common causes were liver-related (149 [32%]), followed by external causes (113 [25%], including trauma, accidents, poisoning, and suicide), and mental health/behavioral disorders (77 [17%]). Similar proportions of females vs males developed a liver-related death (38% vs 29%; *P* = .07), death from an external cause (26% vs 22%; *P* = .32), and mental health/behavioral disorders (16% vs 18%; *P* = .53). However, after considering the competing events of LT and non–liver-related deaths, the cumulative incidence of liver-related mortality at 10 years was 8.2% (95% CI, 6.9%-9.7%) and was higher among females (11.0%; 95% CI, 8.3%-14.2%) compared with males (6.9%; 95% CI, 5.4%-8.6%) (*P* = .01) (eFigure 2B in Supplement 1).

### Association Between Sex and Development of Cirrhosis and Decompensation in AYAs With AH

After excluding AYAs who received a diagnosis of cirrhosis or decompensation, received LT, died, or lost Ontario Health Insurance Plan coverage during the index ED visit or admission (650 [19%]), a further 9% (n = 316) developed these outcomes within 6 months post index (Figure 1). Therefore, 71% (n = 2374) of the cohort was alive and at risk of cirrhosis and decompensation during follow-up (eTable 4 in Supplement 1) and were followed up for a median of 4 (IQR, 2-9) years. Among those, 22% (n = 527) received a diagnosis of cirrhosis after a median of 24 (IQR, 9-50) months, with 42% (n = 220) presenting with decompensation. The most common decompensation events were ascites (88 [40%]) and variceal bleeding (66 [30%]). The cumulative incidence of cirrhosis and/or decompensation at 20 years was 32.5% (95% CI, 29.6%-35.4%), with most developing cirrhosis within the first 5 years after the initial AH presentation (20.5%; 95% CI, 18.8%-22.4%).

When stratified by sex, females were more likely to be diagnosed with cirrhosis and/or decompensation vs males (37% vs 28%; *P* < .001). Of those with cirrhosis, females were more likely to present with decompensation vs males (47% vs 38%; *P* < .001). The cumulative incidence of cirrhosis and/or decompensation at 5 years was 24.7% among females (95% CI, 21.4%-28.1%) vs 18.6% among males (95% CI, 16.5%-20.7%) (*P* < .001) (eFigure 3 in Supplement 1). After multivariate competing risks regression accounting for death and LT as competing events (Table 3), female sex was independently associated with a 47% higher subhazard of cirrhosis and/or decompensation (sHR, 1.47; 95% CI, 1.23-1.76; *P* < .001). Other factors associated with cirrhosis and/or decompensation included residing in the lowest income neighborhoods (sHR, 1.37; 95% CI, 1.01-1.87), older age (per year sHR, 1.04; 95% CI, 1.03-1.06), more comorbidities (sHR, 1.36; 95% CI, 1.01-1.82), and residing in an urban location (sHR, 1.29; 95% CI, 1.02-1.65). The need for hospitalization at index was not associated with the development of liver-related events (sHR, 1.17; 95% CI, 0.91-1.50).

**Table 3.** Competing Risk Regression Analysis Evaluating the Association Between Sex and Cirrhosis and/or Decompensation Among 2374 Adolescents and Young Adults After First Presentation of Alcohol-Associated Hepatitis

Variable	Unadjusted		Adjusted	
	sHR (95% CI)	*P* value	sHR (95% CI)	*P* value
Female vs male sex	1.35 (1.13-1.61)	<.001	1.47 (1.23-1.76)	<.001
Age, y	1.04 (1.03-1.06)	<.001	1.04 (1.03-1.06)	<.001
Type of presentation				
ED only	1 [Reference]	NA	1 [Reference]	NA
Hospital admission	1.09 (0.92-1.31)	.32	1.03 (0.86-1.24)	.73
ECI				
0-2	1 [Reference]	NA	1 [Reference]	NA
≥3	1.49 (1.12-1.98)	.007	1.36 (1.01-1.82)	.04
Urban location	1.32 (1.05-1.68)	.02	1.29 (1.02-1.65)	.04
Recent immigrant/refugee	1.23 (0.97-1.56)	.10	1.17 (0.91-1.50)	.21
Income quintile				
1 (Lowest)	1.39 (1.03-1.87)	.03	1.37 (1.01-1.87)	.04
2	1.32 (0.96-1.83)	.09	1.26 (0.91-1.75)	.16
3	1.45 (1.04-2.02)	.03	1.4 (1.00-1.96)	.05
4	1.29 (0.92-1.81)	.15	1.28 (0.91-1.81)	.15
5 (Highest)	1 [Reference]	NA	1 [Reference]	NA

Abbreviations: ECI, Elixhauser comorbidity index; ED, emergency department; NA, not applicable; sHR, subdistribution hazard ratio.

## Discussion

In this population-based study of 3340 AYAs with AH, we observed that in the general population, the rates of AH have been increasing rapidly, especially among females. Additionally, we highlight a striking sex disparity. Although in absolute numbers more males are affected by AH than females, females have higher rates of liver-related mortality and are at an approximately 50% higher risk of developing cirrhosis than their male counterparts. These data highlight the evolving epidemic of ALD among AYAs occurring in Canada and have broader relevance, given the global nature of increasing harms from alcohol use.

The reasons for increasing prevalence of AH among AYAs are not known; however, we hypothesize several factors may be contributing. Changes to alcohol policy that expand alcohol access are known to be associated with increased harm.^15,16^ During our study, the Ontario government implemented policies that expanded alcohol access.^17,18^ The presence of underlying cofactors for liver disease that have been shown to be associated with a higher risk of AH are increasing among Canadian AYAs, including obesity,^19^ diabetes,^20^ and viral hepatitis.^21^ In our cohort, 8% had viral hepatitis and the incidence was higher among females (10%) than males (7%) while diabetes was present in 6%, which is much higher than the less than 1% prevalence of both viral hepatitis and diabetes in individuals younger than 40 years in the general population.^21^ Changes in the types of alcohol consumed by AYAs may also be a factor. In both the US and Canada,^22^ sales for single-serve flavored beverages, such as ciders and coolers, that contain high amounts of both alcohol and sugar have more than tripled over the past decade. In the US, data suggest that the highest consumers of these beverages are those born after 1980.^23^ Most data on the risk of ALD are based on consumption of either beer, wine, or spirits, all of which have relatively low sugar content. Yet, data have suggested that high fructose consumption also increases the risk of ALD.^24^ Whether the combination of high alcohol and sugar together influence the natural history of ALD is unknown but should be evaluated in future studies, including the impact of comorbid obesity, diabetes, and viral hepatitis.

To our knowledge, there are no data regarding the development of liver-related complications among AYAs after an episode of AH. Most studies evaluating the progression from AH to cirrhosis are from historic, small, older, predominately male cohorts from the 1970s and 1980s with study designs at risk of selection bias and loss to follow-up. In the AYAs cohort in our study, the 5-year overall mortality after AH is approximately 25%, with liver-related causes of death most common, followed by external injury and mental health conditions. It is noteworthy that more than 50% of this cohort had previously contacted health care for mental health conditions, about one-third had presented with substance use within the 2 years before AH presentation, and more than two-thirds had health care encounters related to alcohol that were not AH within the 2 years before AH presentation. This suggests that there are likely missed opportunities to identify those with alcohol use disorder and harmful alcohol use before they progress to AH. Additionally, these results support the need for comprehensive care for managing treatment of patients with AH inclusive of support for liver disease, addictions, and mental health.

Our study provides insights into at-risk populations. We uncovered sex-specific differences in sociodemographic and social determinants of health. Females presenting with AH were more likely to live rurally, in neighborhoods of the lowest income quintile, and have a history of mental health conditions, while males were more likely to be an immigrant or refugee and live in ethnically diverse neighborhoods. These observations emphasize the importance of understanding the social and cultural factors of the population in need of intervention, as these are key for the development and delivery of behavioral therapy for alcohol use disorder programs. The findings also highlight the need to ensure protocols are in place to aid in the recruitment of diverse patient populations into clinical trials for alcohol use disorder and AH that reflect the population’s diversity.

### Strengths and Limitations

Our study has many strengths, including the use of population-level data comprising a diverse population in a contemporary era with longitudinal follow-up and use of validated algorithms to define important liver-related outcomes. However, some limitations should be considered. First, our definition of AH was based on *ICD* codes if listed as the primary diagnosis. Although this has been validated in other data, it has not been validated in ICES. Second, we do not have access to data on treatments for AH, specifically the use of steroids, and therefore how treatment impacts outcomes in this cohort is unknown. However, since steroids for treatment of severe AH are only associated with a short-term mortality benefit,^25^ this is unlikely to affect our results on incident cirrhosis given the follow-up time for this outcome started 6 months after the index presentation to exclude prevalent cirrhosis. Cause-specific mortality data were only available to the end of 2018, including 55% of overall deaths. Therefore, it is unclear whether sex-specific causes of death from 2019 to 2022 remained similar during the COVID-19 pandemic. Third, ICES does not contain data related to the treatment of alcohol use disorder, quantity of alcohol consumption, obesity, race and ethnicity, marital status, or social supports, which may also be associated with the development of AH and our outcomes.

## Conclusions

In this cohort study, rates of AH were observed to increase at concerning rates among AYAs, with females most at risk for liver-related outcomes and death. These data support the need to develop early identification and treatment for those at risk for AH and multidisciplinary management after their first presentation. Furthermore, the increase of AH among AYAs highlights the need to develop clinical trials and observational studies with a sex or gender approach to best support this younger vulnerable population.

## Notes

Supplement 1. **eTable 1.** List and Description of ICES Databases Used **eTable 2.** Codes and Definitions for Variables and Outcomes Used in Study **eTable 3.** Individuals in Ontario Aged 13-39 Years With First Presentation of Alcohol-Associated Hepatitis Without Known History of Cirrhosis, Decompensation, or LT **eTable 4.** Individuals in Ontario Aged 13-39 Years Without History of Cirrhosis, Decompensation, or LT Within 6 Months of First Presentation of Alcohol-Associated Hepatitis **eFigure 1.** Cumulative Incidence of Overall Mortality Among Adolescents and Young Adults With First Presentation of Alcohol-Associated Hepatitis **eFigure 2.** Cause-Specific Mortality Among Adolescents and Young Adults After First Presentation of Alcohol-Associated Hepatitis **eFigure 3.** Cumulative Incidence of Cirrhosis +/− Decompensation Among At-Risk Adolescents and Young Adults After First Presentation of Alcohol-Associated Hepatitis **eMethods.** Demographics and Covariates
eReferences

Supplement 2.
Data Sharing Statement
