# Supplementary material for: Epidemiology and Outcomes of Alcohol-Associated Hepatitis in Adolescents and Young Adults
Source: JAMA Netw Open. 2024 Dec 27;7(12):e2452459. doi: 10.1001/jamanetworkopen.2024.52459 (PMC11681377; doi:10.1001/jamanetworkopen.2024.52459)
Supplement: Supplement 1. — eTable 1. List and Description of ICES Databases Used eTable 2. Codes and Definitions for Variables and Outcomes Used in Study eTable 3. Individuals in Ontario Aged 13-39 Years With First Presentation of Alcohol-Associated Hepatitis Without Known History of Cirrhosis, Decompensation, or LT eTable 4. Individuals in Ontario Aged 13-39 Years Without History of Cirrhosis, Decompensation, or LT Within 6 Months of First Presentation of Alcohol-Associated Hepatitis eFigure 1. Cumulative Incidence of Overall Mortality Among Adolescents and Young Adults With First Presentation of Alcohol-Associated Hepatitis eFigure 2. Cause-Specific Mortality Among Adolescents and Young Adults After First Presentation of Alcohol-Associated Hepatitis eFigure 3. Cumulative Incidence of Cirrhosis +/− Decompensation Among At-Risk Adolescents and Young Adults After First Presentation of Alcohol-Associated Hepatitis eMethods. Demographics and Covariates eReferences [file jamanetwopen-e2452459-s001.pdf]

Supplementary Online Content

Flemming JA, Djerboua M, Chapman O, Ayonrinde O, Terrault NA. Epidemiology and outcomes of alcohol-associated hepatitis in adolescents and younger adults. *JAMA Netw Open*. 2024;7(12):e2452459. doi:10.1001/jamanetworkopen.2024.52459

eTable 1. List and Description of ICES Databases Used

eTable 2. Codes and Definitions for Variables and Outcomes Used in Study

eTable 3. Individuals in Ontario Aged 13-39 Years With First Presentation of Alcohol-Associated Hepatitis Without Known History of Cirrhosis, Decompensation, or LT

eTable 4. Individuals in Ontario Aged 13-39 Years Without History of Cirrhosis, Decompensation, or LT Within 6 Months of First Presentation of Alcohol-Associated Hepatitis

eFigure 1. Cumulative Incidence of Overall Mortality Among Adolescents and Young Adults With First Presentation of Alcohol-Associated Hepatitis

eFigure 2. Cause-Specific Mortality Among Adolescents and Young Adults After First Presentation of Alcohol-Associated Hepatitis

eFigure 3. Cumulative Incidence of Cirrhosis +/- Decompensation Among At-Risk Adolescents and Young Adults After First Presentation of Alcohol-Associated Hepatitis

eMethods. Demographics and Covariates

eReferences

This supplementary material has been provided by the authors to give readers additional information about their work.

© 2024 Flemming JA et al. *JAMA Network Open.*

**eTable 1.** List and description of ICES databases used.	
Immigration, Refugees and Citizenship Canada's Permanent Residence Database (CIC-IRCC)	CIC-IRCC contains individual-level information for all immigration application records for people who landed in Ontario from 1985 to 2020, including country of origin, level or education, and landing date. CIC-IRCC was used to establish recent immigrant or refugee status in adolescents and young adults with AH presentation from 2002 to 2021.
Canadian Institute for Health Information Discharge Abstract Database (DAD)	DAD has patient-level information for all inpatient hospital admissions in Ontario from 1988-onwards, including diagnoses, procedures, and provider services. DAD was used to identify initial AH presentation in adolescents and young adults, as well as covariates, cirrhosis and decompensation outcomes, and healthcare utilization patterns.
ICES Physician Database (IPDB)	IPDB has individual-level information for all physicians in Ontario, including demographics, specialty, location, and physician activity load. IPDB was used to establish healthcare utilization patterns, namely consultations with internal medicine, gastroenterology, psychiatry specialists.
National Ambulatory Care Reporting system (NACRS)	NACRS has patient-level for all ambulatory care and emergency department visits from 2000 onwards, including diagnoses, procedures, and provider services. NACRS was used to identify initial AH presentation in adolescents and young adults, as well as covariates and healthcare utilization patterns.
Ontario Health Insurance Plan Physician Claims Database (OHIP)	OHIP has all claims made by physicians and other healthcare providers for services and procedures covered by OHIP from 1991-onwards. OHIP was used to establish

© 2024 Flemming JA et al. *JAMA Network Open.*

	covariates, healthcare utilization patterns, and the cirrhosis outcomes.
Ontario Laboratory Information System (OLIS)	OLIS contains order requests and test results from select inpatient and outpatient laboratories in Ontario. Serum creatinine, total bilirubin, international normalized ratio for prothrombin time, and sodium tests from OLIS were used to calculate MELD-Na score for adolescents and young adults at first AH presentation.
Ontario Mental Health Reporting System (OMHRS)	OMHRS has patient-level information for all inpatient admissions to mental health institutions in Ontario, including diagnoses (for both mental and physical health),
Ontario Marginalization Index (ONMARG)	ONMARG contains multiple measures of health and social well-being for population groups and geographics areas. Four of the ONMARG quintile measures (material deprivation, ethnic diversity, residential instability, and dependency) were used in this study to describe social determinants of health in the study population.
Office of the Registrar General-Deaths (ORGD)	ORGD contains information on all deaths registered in Ontario, including cause of death, from 1990-2018. ORGD was used to establish cause-specific death in the study population, including liver-related, cancer-related, mental health-related, and death related to external causes.
Postal Code Conversion Files (PCCF)	PCCF is a file from Statistics Canada and Canada Post Corporation that contains census and geographic information for 6-character Canadian postal codes. PCCF linked to individual postal code provided by RPDB to establish individual's type of residence (rural versus urban residence) and neighbourhood-level income quintile.
Public Health Ontario Laboratories (PHOL)	PHOL contains information on all hepatitis B and C tests in Ontario from 1997-2018, including antibodies, RNA, DNA, surface antigens, and genotyping. PHOL was used to establish viral hepatitis history in adolescents and young adults prior to first AH presentation.
Registered Persons Database (RPDB)	RPDB includes demographic information for all individuals in Ontario eligible for OHIP.

© 2024 Flemming JA et al. *JAMA Network Open.*

	RPDB was used for the main exposure of interest in this study, sex. RPDB was also used to describe additional demographics including age and postal code, as well as the death date which was used to develop the mortality outcomes.

© 2024 Flemming JA et al. *JAMA Network Open.*

**eTable 2.** Codes and definitions for variables and outcomes used in study.		
**Concept**	**Data Sources**	**Definition**
**Study Population**		
Alcohol-associated hepatitis	DAD, NACRS	ICD-9: 571.1<br>ICD-10: K70.1
**Covariates**		
History of ED or inpatient visit for alcohol	DAD, NACRS	ICD-9: 291, 303, 3050<br>ICD-10: F10
	OMHRS	DSM-5: 291, 303, 3050<br>ICD-10: F10
History of mental illness (including psychiatric and mood disorder)	DAD, NACRS	ICD-9: 295, 296, 297, 298, 3000, 3002, 3003, 3004, 3010, 3012, 3014, 3015, 3016, 3017, 3018, 3019, 3071, 3075, 3083, 3090, 3092, 3093, 3094, 3098, 3099, 311<br>ICD-10: F060 F061 F062 F063 F064 F070 F20 F21 F22 F23, F24, F25, F28, F29, F30, F31, F32, F33, F34, F38, F39, F40, F41, F42, F43, F50, F60, F61, F62
	OMHRS	DSM-5: 29381, 29382, 29383, 295, 296, 297, 298, 3000, 3002, 3003, 3004, 3010, 30113, 3012, 3014, 3015, 3016, 3017, 3018, 3019, 3071, 30750, 30751, 3083, 3090, 30924, 30928, 3093, 3094, 30981, 30989, 3099, 311<br>ICD-10: F060 F061 F062 F063 F064 F070 F20 F21 F22 F23, F24, F25, F28, F29, F30, F31, F32, F33, F34, F38, F39, F40, F41, F42, F43, F50, F60, F61, F62
	OHIP	ICD-9 (3-digit): 295, 296, 297, 298, 300, 301, 307, 309, 311
History of substance use	DAD, NACRS	ICD-9: 2920, 2921, 2922, 2928, 2929, 3040, 3041, 3042, 3043, 3044, 3045, 3046, 3047, 3048, 3049, 3052, 3053, 3054, 3055, 3056, 3057, 3058, 3059<br>ICD-10: F11-F19
	OMHRS	DSM-5: 2920, 2921, 2922, 2928, 2929, 30400, 30410, 30420, 30430, 30440, 30450, 30460, 3047, 30480, 30490, 30520, 30530, 30540, 30550, 30560, 30570, 3058, 30590<br>ICD-10: F11-F19

© 2024 Flemming JA et al. *JAMA Network Open.*

	OHIP	ICD-9 (3-digit): 304, 292
Comorbid viral hepatitis	DAD, NACRS	ICD-9: 0703, 0705<br>ICD-10: B180, B181, B182
	OHIP	ICD-9 (3-digit): 070, 964
	OLIS	Tests with following LOINCs for HBV surface antigen (sAg), HBV DNA, or HCV RNA and positive result.<br><br>Hepatitis B:<br>LOINC = 5195-3, 65633-0, 5196-1, 7905-3, 13126-8, 29610-3, 42595-9, 11258-1, 20442-0<br><br>Hepatitis C:<br>LOINC =11259-9, 29609-5, 10676-5, 20571-6, 20416-4, 32286-7
	PHOL	Tests for HBV sAg, HBV DNA, or HCV RNA with "Reactive" or "Detected" result.
**Outcomes**		
Cirrhosis	DAD	ICD-9: 571.2, 571.5, 456.1<br>ICD-10: K70.3, K71.7, K74.6, I85.9, I98.21
	OHIP	ICD-9 (3-digit): 571
Decompensated cirrhosis	DAD	ICD-9: 572.2, 572.4, 456.0, 456.8, 570, 572.8, 456.2, 782.4, 789.5<br>ICD-10: K72.0, K72.1, K72.9, K76.7, R17, R18, I85.0, I86.4, I98.2, I98.20, I98.3
**Cause-specific death**		
Liver-related (liver disease, viral hepatitis, or hepatobiliary malignancies)	ORGD	Liver disease:<br>ICD-9: 570, 571, 572, 573, 4560, 4561, 4562, 4568<br>ICD-10: K70, K71, K72, K73, K74, K75, K76, I85, I864, I982, I983<br><br>Viral hepatitis:<br>ICD-9: 070<br>ICD-10: B15, B16, B17, B18, B19<br><br>Hepatobiliary cancer:<br>ICD-9: 155<br>ICD-10: C22
Cancer-related	ORGD	ICD-10: C00-D48 (excluding C22)
Mental health-related	ORGD	ICD-10: F00-F99

© 2024 Flemming JA et al. *JAMA Network Open.*

External causes of death (including injuries, accidents, and poisoning)	ORGD	ICD-10: V01-Y98
**Other variables**		
Liver transplant	CORR	In RECIPIENT_TREATMENT dataset: Treatment_code=171, 181 AND Transplanted_organ_type_code[1,2,3] = 20 (Liver), 21 (Liver-left lobe), 22 (Liver-right lobe), 23 (Liver-lateral segment), 29 (Liver-two[from conversion])
	DAD	CCP: 6240, 6241, 6249<br>CCI: 1.OA.85
	OHIP	Fee Codes: S294, S266
Abbreviations: DSM-V = Diagnostic and Statistical Manual of Mental Disorders, 5th Edition; ICD-9 = International Classification of Diseases, 9th Revision (1988-2001); ICD-10 = International Classification of Diseases, 10th Revision (2002 onwards), CCI = Canadian Classification of Health Interventions (2002 onwards), CCP = Canadian Classification of Diagnostic, Therapeutic, and Surgical Procedures (1988-2002); LOINC = Logical Observation Identifiers Names and Codes.		

© 2024 Flemming JA et al. *JAMA Network Open.*

**eTable 3:** Individuals in Ontario aged 13-39 years with first presentation of alcohol-associated hepatitis without known history of cirrhosis, decompensation or LT: 2002-2021 stratified by hospital admissions vs. ED presentation only (n=3,340).		
	**Hospital Admission n=2,374**	**ED presentation only n=917**
**Age at presentation**, median (IQR)	33 (29-36)	32 (27-36)
**Female sex,** n (%)	879 (36)	311 (34)
^**Rural residence**, n (%)	357 (15)	227 (25)
Missing, n (%)	10 (<1)	6 (<1)
#**Recent immigrant/refugee**, n (%)	330 (14)	104 (11)
***History of ED/inpatient visit for alcohol,** n (%)	1,772 (73)	593 (65)
***History of mental illness,** n (%)	1,417 (58)	495 (54)
***History of substance use,** n (%)	841 (35)	295 (32)
**Viral hepatitis,** n (%)	196 (8)	86 (9)
**ECI,** n (%)		
0-2	2,212 (91)	861 (94)
3+	211 (9)	56 (6)
****MELD-Na,** median (IQR)	16 (10-23)	9 (7-13)
<10	227 (9)	92 (10)
10-20	392 (16)	47 (5)
>20	306 (13)	15 (2)
**Income quintile**, n (%)		
1 (lowest)	754 (31)	326 (36)
2	478 (20)	189 (21)
3	411 (17)	142 (15)
4	405 (17)	132 (14)
5 (highest)	347 (14)	115 (13)
Missing	28 (1)	13 (1)
**Material Deprivation quintile**, n (%)		
1 (lowest)	357 (15)	122 (13)
2	360 (15)	121 (13)
3	415 (17)	132 (14)
4	483 (20)	180 (20)
5 (highest)	642 (26)	264 (29)
Missing	166 (7)	98 (11)
**Ethnic Diversity quintile**, n (%)		
1 (lowest)	372 (15)	193 (21)
2	376 (16)	155 (17)
3	457 (19)	159 (17)
4	460 (19)	137 (15)
5 (highest)	592 (24)	175 (19)
Missing	166 (7)	98 (11)
**Residential Instability quintile**, n (%)		
1 (lowest)	345 (14)	109 (12)
2	328 (14)	120 (13)

© 2024 Flemming JA et al. *JAMA Network Open.*

3	390 (16)	149 (16)
4	458 (19)	160 (17)
5 (highest)	736 (30)	281 (31)
Missing	166 (7)	98 (11)
**Follow-up time,** median years (IQR)	5 (2-9)	7 (3-12)
**Died anytime during follow-up, n (%)**	660 (27)	184 (20)
**Liver transplant,** n (%)	17-22 (<1)	<6 (<1)

^ rural defined as location with <10,000 inhabitants; # immigrant or refugee who landed after 1985; * history within two-years of alcohol-associated hepatitis presentation. **MELD-Na if available +/- 7 days from presentation, missing values for n=2,261 (68%). IQR: interquartile range; ED: emergency department

© 2024 Flemming JA et al. *JAMA Network Open.*

**eTable 4:** Individuals in Ontario aged 13-39 years without history of cirrhosis, decompensation or LT within 6 months of first presentation of alcohol-associated hepatitis 2002-2021 stratified by sex (n=2,374).		
	**Females**<br>**n=793**	**Males**<br>**n=1,581**
**Age at presentation**, median (IQR)	31 (27-35)	32 (28-36)
^**Rural residence**, n (%)	194 (24)	263 (17)
Missing, n (%)	<6 (<1)	9 (0.57)
#**Recent immigrant/refugee**, n (%)	47 (6)	255 (16)
***History of ED/inpatient visit for alcohol,** n (%)	577 (73)	1,135 (72)
***History of mental illness,** n (%)	513 (65)	857 (54)
***History of substance use,** n (%)	289 (36)	563 (36)
**Viral hepatitis,** n (%)	62 (8)	117 (7)
**ECI,** n (%)		
0-2	727 (92)	1,458 (92)
3-5	60 (8)	108 (7)
6+	6 (<1)	15 (<1)
****MELD-Na,** median (IQR)	11 (8-16)	12 (9-16)
<10	106 (13)	177 (11)
10-20	111 (14)	195 (12)
>20	30 (4)	55 (4)
**Income quintile**, n (%)		
1 (lowest)	288 (36)	482 (30)
2	140 (18)	341 (22)
3	119 (15)	273 (17)
4	131 (16)	240 (15)
5 (highest)	104 (13)	222 (14)
Missing	11 (1)	23 (1)
**Material Deprivation quintile**, n (%)		
1 (lowest)	115 (15)	235 (15)
2	101 (13)	213 (13)
3	111 (14)	275 (17)
4	144 (18)	326 (21)
5 (highest)	226 (28)	411 (26)
Missing	96 (12)	121 (8)
**Ethnic Diversity quintile**, n (%)		
1 (lowest)	135 (17)	281 (18)
2	142 (18)	220 (14)
3	160 (20)	286 (18)
4	134 (17)	269 (17)
5 (highest)	126 (16)	404 (26)
Missing	96 (12)	121 (8)
**Residential Instability quintile**, n (%)		
1 (lowest)	86 (11)	228 (14)
2	83 (10)	233 (15)
3	128 (16)	247 (16)

© 2024 Flemming JA et al. *JAMA Network Open.*

4	153 (19)	267 (17)
5 (highest)	247 (31)	485 (31)
Missing	96 (12)	121 (8)
**Follow-up time,** median years (IQR)	5 (3-10)	6 (3-12)
**Died anytime during follow-up, n (%)**	140 (18)	326 (21)
**Liver transplant,** n (%)	<6 (<1)	<6 (<1)

^ rural defined as location with <10,000 inhabitants; # immigrant or refugee who landed after 1985; * history within two-years of alcohol-associated hepatitis presentation. **MELD-Na if available +/- 7 days from presentation, missing values for n=1,700 (72%). IQR: interquartile range; ED: emergency department

© 2024 Flemming JA et al. *JAMA Network Open.*

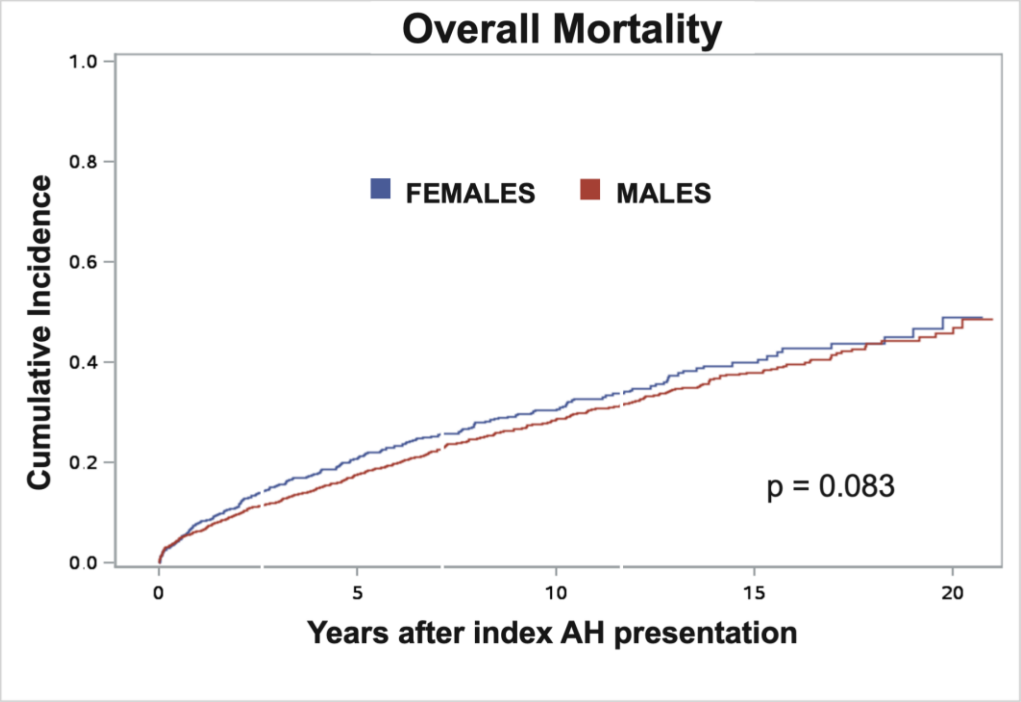

eFigure 1: Cumulative incidence of overall mortality among adolescents and young adults with first presentation of alcohol-associated hepatitis in Ontario 2002-2021 stratified by sex (n=3,340).

© 2024 Flemming JA et al. *JAMA Network Open.*

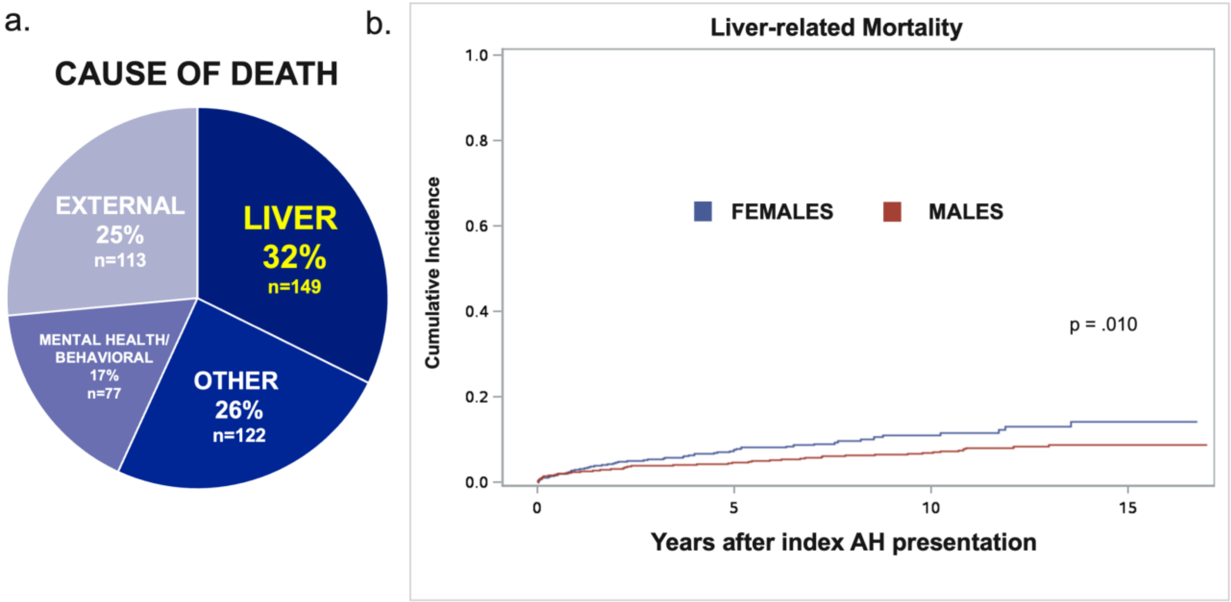

eFigure 2: Cause specific mortality among adolescents and young adults after first presentation of alcohol-associated hepatitis (AH) in Ontario 2002-2018 (n=461 deaths).

© 2024 Flemming JA et al. *JAMA Network Open.*

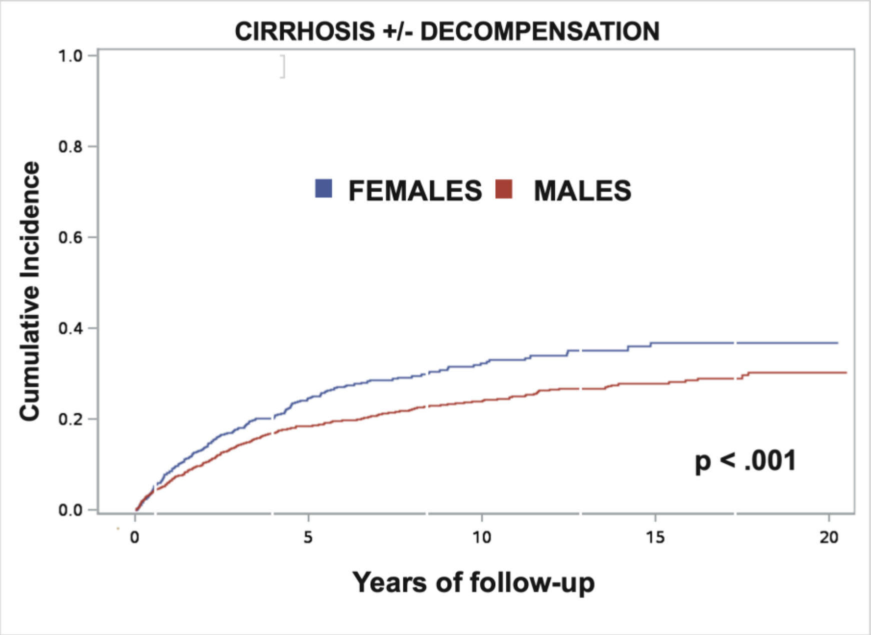

eFigure 3: Cumulative incidence of cirrhosis +/- decompensation among at risk adolescents and young adults after first presentation of alcohol-associated hepatitis in Ontario 2002-2021 (n=2,374).

© 2024 Flemming JA et al. *JAMA Network Open.*

eMethods. Demographics and covariates

sTable 2 has full details on the definitions of study demographics and co-variates. In summary, age at first presentation for AH was calculated using the individual's birth date in the RPDB. Rural versus urban residence was defined based on communities +/- 10,000 inhabitants. Income quintile was based on linkage of the individual's postal code in RPDB to the PCCF for the Census-based relative household income at the dissemination area-level. Immigrant/refugee status since 1985 was defined by linking to the IRCC-CIC. Four area-level quintile measurements representing social determinants of health were defined by linkage to the ON-MARG database including material deprivation, ethnic diversity, residential instability, and dependency. ON-MARG's quintile scales ranged from 1 (individual resides in an area that is the least marginalized for that dimension) to 5 (area is the most marginalized for that dimension). Healthcare contact within two years prior to index was identified and included contact for harmful alcohol use not related to AH, history of substance use other than alcohol, history of severe mental illness (including psychotic and mood disorders), and the Elixhauser Comorbidity Index (ECI)[1] was used to summarize co-morbid illness. We identified patients with viral hepatitis B and C through positive viral serology tests for hepatitis B and C in OLIS and PHOL, and diagnostic codes in DAD, NACRS, or OHIP. Among those with available laboratory data, the Model for End-Stage Liver Disease-Sodium score (MELD-Na) was calculated using serum creatinine, total bilirubin, international normalized ratio for prothrombin time, and sodium that were the closest within 7 days of the AH presentation.[2] Outpatient consultations were identified through OHIP physician claims linked to the ICES Physicians Database to establish the main medical specialty associated with the physician billing.

© 2024 Flemming JA et al. *JAMA Network Open.*

eReferences

1. Van Walraven C, Austin PC, Jennings A, Quan H, Forster AJ. A modification of the elixhauser comorbidity measures into a point system for hospital death using administrative data. *Medical Care*. 2009;47(6):626-633. doi:10.1097/MLR.0b013e31819432e5
2. Nagai S, Chau LC, Schilke RE, et al. Effects of Allocating Livers for Transplantation Based on Model for End-Stage Liver Disease–Sodium Scores on Patient Outcomes. *Gastroenterology*. 2018;155(5):1451-1462.e3. doi:10.1053/j.gastro.2018.07.025

List of abbreviations in the manuscript

ALD: alcohol-associated liver disease

AH: alcohol-associated hepatitis

AYA**:** adolescent and young adult

CI: confidence interval

CIF: cumulative incidence function

ED: emergency department

ICD: international classification of diseases

IQR: interquartile range

LT: liver transplant

MELD-Na: Model for end stage liver disease

OHIP: Ontario health insurance plan

PY: person-years

RPDB: registered persons database

RR: rate ratio

sHR: sub distribution hazard

© 2024 Flemming JA et al. *JAMA Network Open.*
