# Supplementary material for: Epidemiology and Outcomes of Alcohol-Associated Hepatitis in Adolescents and Young Adults
Source: JAMA Netw Open. 2024 Dec 27;7(12):e2452459. doi: 10.1001/jamanetworkopen.2024.52459 (PMC11681377; doi:10.1001/jamanetworkopen.2024.52459)
Supplement: Supplement 2. — Data Sharing Statement [file jamanetwopen-e2452459-s002.pdf]

Data Sharing Statement

Flemming. Epidemiology and Outcomes of Alcohol-Associated Hepatitis in Adolescents and Young Adults. *JAMA Netw Open*. Published December 27, 2024.
doi:10.1001/jamanetworkopen.2024.52459

Data
Data available: Yes
Data types: Deidentified participant data
How to access data: The dataset from this study is held securely in coded form at ICES. While legal data sharing agreements between ICES and data providers (e.g., healthcare organizations and government) prohibit ICES from making the dataset publicly available, access may be granted to those who meet pre-specified criteria for confidential access, available at www.ices.on.ca/DAS (email: das@ices.on.ca). The full dataset creation plan and underlying analytic code are available from the authors upon request, understanding that the computer programs may rely upon coding templates or macros that are unique to ICES and are therefore either inaccessible or may require modification.
When available: With publication

Supporting Documents
Document types: None

Additional Information
Who can access the data: researchers whose proposed use of the data has been approved
Types of analyses: For a specified purpose
Mechanisms of data availability: The dataset from this study is held securely in coded form at ICES. While legal data sharing agreements between ICES and data providers (e.g., healthcare organizations and government) prohibit ICES from making the dataset publicly available, access may be granted to those who meet pre-specified criteria for confidential access, available at www.ices.on.ca/DAS (email: das@ices.on.ca). The full dataset creation plan and underlying analytic code are available from the authors upon request, understanding that the computer programs may rely upon coding templates or macros that are unique to ICES and are therefore either inaccessible or may require modification.
